# Surgical Strategy for Pediatric Liver Tumors Involving the Hepatic Venous Confluence and the Inferior Vena Cava

**DOI:** 10.1245/s10434-025-17245-5

**Published:** 2025-03-26

**Authors:** Juri Fuchs, Lucas Rabaux-Eygasier, Geraldine Hery, Virginie Fouquet, Florent Guerin, Stephanie Franchi-Abella, Sophie Branchereau

**Affiliations:** 1 Department of General, Visceral, Pediatric and Transplantation Surgery, Heidelberg University Hospital, Heidelberg, Germany; 2 Department of Pediatric Surgery, Hôpital Kremlin-Bicêtre, APHP, University of Paris-Saclay, Paris, France; 3 Department of Pediatric Radiology, Hôpital Kremlin-Bicêtre, APHP, University of Paris-Saclay, Paris, France

**Keywords:** Pediatric liver tumors, Pediatric hepatectomy, Hepatoblastoma

## Abstract

**Background:**

Pediatric liver tumors presenting as centrally located masses with contact to or even invasion of all three hepatic veins (HVs) and the inferior vena cava (IVC) present significant surgical challenges. While liver transplantation may be indicated in truly unresectable tumors, extended liver resection with vascular reconstruction can be an organ-preserving alternative.

**Objective:**

This study aimed to present a reference center’s strategy for children with liver tumors involving the hepatic venous confluence or the retrohepatic IVC who underwent extended liver resection with vascular reconstruction.

**Methods:**

All pediatric patients undergoing major hepatectomy with reconstruction of an HV or the IVC over a 10-year study period were included. Preoperative imaging, surgical techniques, and short- and long-term postoperative data were analyzed.

**Results:**

From a total of 125 pediatric major hepatectomies, 17 children (15 hepatoblastoma, two undifferentiated embryonal sarcoma) underwent liver resection with vascular reconstruction of an HV or the IVC. In nine cases an HV was reconstructed, and in eight children, a partial resection of the IVC was performed. Total vascular exclusion of the liver was applied in 16/17 cases. No 90-day postoperative mortality, no major postoperative complication, and no local relapse occurred; 16/17 patients are alive without relapse at a median follow-up of 44 months (range 19–111).

**Conclusion:**

This is the largest single-center series to report major hepatectomies with HV or IVC reconstruction in children. In specialized centers, these complex procedures are associated with excellent outcomes. Successful tumor resection can be achieved in selected cases even in locally advanced tumor stages.

**Supplementary Information:**

The online version contains supplementary material available at 10.1245/s10434-025-17245-5.

For children diagnosed with malignant liver tumors, the stakes are high: the disease is life-threatening, yet the goal of treatment is not just survival but a future free from the burdens of compromised organ function or the long-term effects of radical treatments. Therefore, a central aspect of pediatric liver tumor surgery is finding a balance between surgical risks, radicality, and the preservation of organ function.

Finding this balance is complicated by the frequently encountered manifestation of liver tumors in children as large, centrally located masses, in contact with all three hepatic veins (HVs) and the inferior vena cava (IVC).^1–3^ While liver transplantation may be indicated for cases deemed unresectable due to extensive vascular involvement or panhepatic extension,^4^ this approach is not without its drawbacks, particularly high postoperative morbidity and lifelong immunosuppression.^5–7^ At our center, both resection and transplantation are performed for pediatric liver tumors, aiming to provide the best-suited, tailored treatment for every individual child. In our experience, extended liver resection accepting minimal margins and the potential necessity of a partial resection and reconstruction of an HV or the IVC, is a viable, organ-preserving alternative for certain high-risk patients. The technical demands of such procedures are significant, requiring meticulous planning and profound experience to ensure the safe removal of the tumor with reconstruction of vital vasculature to maintain hepatic function. Mostly reported in adult liver surgery,^8^ such procedures have only rarely been described in children thus far.^1,9,10^ This study delves into a decade of experience with pediatric liver tumors at a tertiary care center specialized in pediatric hepatobiliary surgery, focusing on all consecutive pediatric patients who have undergone major hepatectomy with concurrent reconstruction of an HV or the IVC. Through the lens of these procedures, we explore an organ-preserving strategy for locally advanced liver tumors in children. The feasibility, safety, and outcomes of vascular reconstructions in pediatric liver tumor surgery are analyzed. The goal was to not only highlight the potential for organ preservation in these cases but to also contribute valuable insights into the differences in pediatric hepatobiliary surgery compared with these procedures in adults. By sharing our center’s experience, we aimed to underscore the importance of surgical expertise in pediatric liver tumor treatment to provide tailored therapies and achieve favorable outcomes for these young patients.

## Patients and Methods

### Study Design and Inclusion Criteria

This was a retrospective series of all consecutive pediatric patients undergoing major partial hepatectomy between 1 September 2013 and 31 December 2023 at the Department of Pediatric Surgery of the University Hospital Paris-Bicêtre. The study was reported according to the STROBE (Strengthening the Reporting of Observational Studies in Epidemiology) recommendations^11^ (the STROBE checklist is provided in electronic supplementary material 1). The conduct of this study was approved by the Institutional Review Board of the Medical Faculty of the University Paris-Saclay. All patients <18 years of age who underwent major partial hepatectomy (>3 segments) with concurrent reconstruction of an HV and/or the IVC were included from the institutional liver surgery registry. Patients with supradiaphragmatic extension and tumor thrombus in the right atrium who underwent combined hepatic and cardiac surgery were excluded from this series. Patients were followed postoperatively according to the department’s protocol, including biochemical liver function and cholestasis tests, abdominal ultrasound, and liver elastography.

### Applied Definitions

The Brisbane 2000 terminology for liver resections was applied to define the type of hepatectomy performed,^12^ and the PRETEXT/POST-TEXT staging for pediatric liver tumors was used for tumor staging.^13^ All postoperative events that occurred within 90 days after surgery were classified according to the Clavien–Madadi classification (CMC) for postoperative adverse events in pediatric surgery.^14^ Medians with interquartile ranges were calculated for non-normally distributed variables, and means with standard deviations were calculated for normally distributed variables. Absolute frequencies were expressed as percentages where appropriate.

### Surgical Strategy and Technique

All cases of children with liver tumors referred to our center were discussed in an interdisciplinary conference including pediatric surgeons, radiologists, and oncologists. The final decision on liver resection or liver transplantation was usually made after three to five chemotherapy cycles, depending on the protocol. A meticulous analysis of the vascular anatomy and the extent of tumor involvement was performed based on the latest preoperative sectional imaging. In addition, all patients underwent abdominal ultrasound by a specialized pediatric radiologist on the day before surgery to specifically evaluate venous involvement and patency. Our center’s criteria for surgical exploration with the aim of partial hepatectomy were (1) at least one anatomical sector free of tumor or one sector only marginally affected; (2) at least one patent first-order HV; and (3) if the tumor had contact with the IVC, resection seemed possible without necessity of total IVC replacement.

Patients with tumors involving all liver sectors (POST-TEXT IV) were listed for liver transplantation if no contraindications were present. Moreover, most PRETEXT IV F+ (multifocal) tumors were considered indications for liver transplantation at our center if no contraindications for transplantation (e.g. irresectable/persistent pulmonary metastasis) were present. In very few selected cases of PRETEXT IV F+ with a maximum of three lesions that were amenable to resection by partial anatomic hepatectomy with up to two additional small wedge resections after good response to neoadjuvant chemotherapy, this approach was considered an alternative to transplantation. Of the total of 125 patients undergoing resection for hepatoblastoma during the observation period of this study, two children with PRETEXT IV F+ tumor and no metastasis underwent liver resection.

Preoperative evaluation included assessment of portal vein involvement. Clear signs of tumor invasion of both main portal vein branches, i.e. total obstruction of tumor thrombosis, were considered a contraindication for resection. However, close tumor contact with the main portal vein branches was not deemed prohibitive for surgery if patency was confirmed. In this cohort, no cases required portal vein resection or reconstruction due to tumor involvement. Based on a special agreement with the ‘Agence de la biomédecine’ (French agency for organ donation), patients listed for liver transplantation for irresectable liver tumors were granted special priority access to deceased donor organs, with a median waitlist time of 15 days (range 3–26) at our institution for these patients. For cases considered suited for surgical exploration with the aim of resection, it is our center’s strategy to accept minimal resection margins (<1 mm) in certain cases of locally advanced tumors to avoid liver transplantation for patients who can be successfully treated with liver resection.

All operations were performed by a team consisting of two experienced pediatric hepatobiliary surgeons (VF, FG, GH, SB). The liver was completely mobilized. The hepatic pedicle as well as the supra- and infrahepatic IVC were encircled to prepare a total vascular exclusion (TVE) of the liver. Intraoperative ultrasound was not routinely used. The transection plane was marked with electrocautery based on preoperatively defined anatomic landmarks. In particular, in cases of left trisectionectomy, intraoperative ultrasound was used for identification of the right HV and to delineate the resection line. Transection was always started without any inflow occlusion. When approaching the hepatic venous confluence or when the tumor was invading the retrohepatic IVC, TVE was applied. The resection and reconstruction of major vessels was finished under TVE. Postoperatively, patients were transferred to the intermediate care unit. Routine abdominal ultrasound was performed on postoperative days 1 and 3, and once before discharge home, with Doppler ultrasound of the hepatic vasculature.

## Results

### Study Cohort and Patient Characteristics

**Fig. 1:**
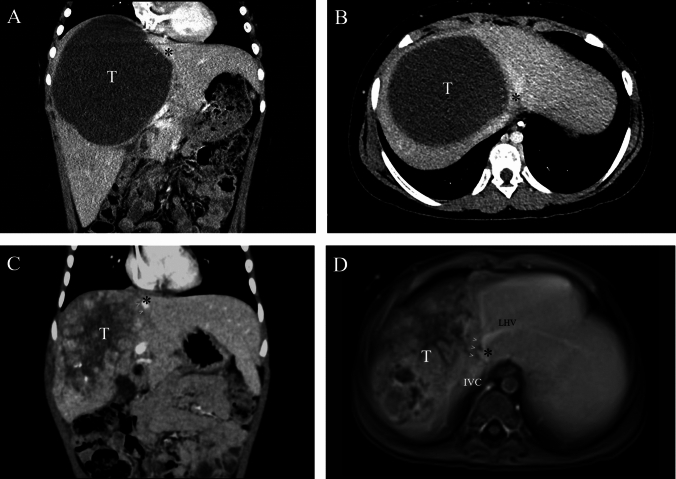
**a, b** CT scan showing the coronal and axial view of patient 3; > arrows showing tumor contact. **c, d** CT scan (coronal) and MRI (axial) of patient 14; > arrows showing tumor contact. *CT* computed tomography, *MRI* magnetic resonance imaging, *T* tumor, *IVC* inferior vena cava, *LHV* left hepatic vein, * common trunk of the middle and left hepatic veins

Of a total of 125 children who underwent major hepatectomy during the observation period, 17 (14%) patients had reconstruction of the IVC or an HV. During the observation period, 18 children underwent liver transplantation for unresectable liver malignancy at our institution (2 children with primary liver angiosarcoma, 16 with hepatoblastoma). Diagnoses in the 17 patients with vascular reconstruction included hepatoblastoma in 14 patients, undifferentiated embryonal liver sarcoma in 2 patients, and hepatocellular neoplasm not otherwise specified (HCN-NOS) in one patient. Median age at surgery was 2 years and 1 month (range 8 months–14 years). Patients 11 and 16 were initially planned to undergo liver transplantation. In patient 11, initial imaging suggested obstruction of all three HVs, however restaging showed a patent right HV. Patient 16 had a multifocal tumor with pulmonary metastases, and underwent two-stage removal of bilateral lung metastases. After neoadjuvant chemotherapy, an initially suspected lesion in the right posterior sector was no longer visible, and a left trisectionectomy was planned. All but one patient had POST-TEXT 3 tumors after neoadjuvant chemotherapy. These patients had either tumors in close contact with all three HVs and/or involvement of the retrohepatic IVC. One patient had a PRETEXT 2/POST-TEXT 2 hepatoblastoma and tumor invasion of the retrohepatic IVC (V+). Seven patients had a tumor thrombus in a major vessel (first-order HV and/or IVC). Table 1 shows an overview of the patients’ baseline characteristics, and Fig. 1 shows sectional imaging of two example cases.

**Table 1:** Patient baseline characteristics

Patient no.	Age	Sex	Diagnosis	Treatment protocol/preoperative chemotherapy	Pretext/post-text	Tumor location/ affected segments	Major vascular* involvement	Tumor thrombosis major vessel*
1	4 y 7 m	F	Hepatoblastoma	SIOPEL-4/3 cycles	3/3	4, 5, 8	Contact to all three HVs	–
2	1 y 3 m	M	Hepatoblastoma	SIOPEL-3/5 cycles	3/3	1, 4, 5, 6, 7, 8	Retrohepatic IVC	Retrohepatic IVC
3	10 y 7 m	F	Undifferentiated embryonal sarcoma	IVADO/4 cycles	3/3	4, 5, 8	Contact to all three HVs	–
4	10 m	F	Hepatoblastoma	SIOPEL-6/4 cycles	3/3	1, 4, 5, 7, 8	Contact to all three HVs	–
5	8 y 11 m	M	Hepatoblastoma	SIOPEL-3/6 cycles	3/3	4, 5, 8	IVC around the orifice of the right HV	–
6	2 y 7 m	M	Hepatoblastoma	SIOPEL-4/2 cycles	2/2	7, 8	Retrohepatic IVC	–
7	1 y	M	Hepatoblastoma	SIOPEL-6/4 cycles	3/3	4, 5, 6, 7, 8	Contact to all three HVs	Middle HV
8	1 y 8 m	M	Hepatoblastoma	SIOPEL-3/5 cycles	3/2	5, 6, 7, 8	IVC around the orifice of the right HV	Right HV
9	2 y 4 m	F	Hepatoblastoma	SIOPEL-3/4 cycles	3/3	1, 7, 8	Retrohepatic IVC	–
10	11 y 11 m	F	Hepatoblastoma	SIOPEL-4/4 cycles	3/3	4, 5, 6, 7, 8	Contact to all three HVs	Middle HV
11	1 y 2 m	M	Hepatoblastoma	SIOPEL-4/3 cycles	3/3	2, 3, 4, 5	Contact to all three HVs	–
12	14 y 2 m	F	Undifferentiated embryonal sarcoma	IVADO/4 cycles	3/3	4, 7, 8	Contact to all three HVs	Right and middle HV
13	2 y 1 m	F	Hepatoblastoma	PHITT C/3 cycles	3/3	1, 4, 5, 6, 7, 8	Retrohepatic IVC	–
14	1 y 10 m	M	Hepatoblastoma	PHITT B/4 cycles	3/3	4, 5, 7, 8	Contact to all three HVs	–
15	8 m	M	Hepatoblastoma	PHITT C/4 cycles	3/3	1, 4, 5, 7, 8	Contact to all three HVs	–
16	2 y 2 m	M	HCN-NOS	PHITT D/3 cycles	4/3	2, 3, 4, 5, 7, 8	Retrohepatic IVC	Right HV
17	1 y 5 m	F	Hepatoblastoma	PHITT C / 3 cycles	3/3	1, 4, 5, 6, 7, 8	Retrohepatic IVC	Retrohepatic IVC

*HV* hepatic vein, *IVC* inferior vena cava, *y* years, *m* months, *F* female, *M* male, *HCN-NOS* hepatocellular neoplasm not otherwise specified, *IVADO* regimen with ifosfamide, vincristine, actinomycin D, and doxorubicin, *POST-TEXT* post-treatment extent of tumor, *PRETEXT* pretreatment extent of tumor, *SIOPEL* Societé Internationale Oncologie Pediatrique and Epitelial Liver Tumor Study Group, *PHITT* Paediatric Hepatic International Tumour Trial

*Major vessel defined as retrohepatic inferior vena cava, first order hepatic vein or first or portal vein branch

### Performed Procedures and Intraoperative Data

The most commonly performed procedure was right trisectionectomy (*n* = 10) in seven cases with resection of segment I. The other procedures were four right hemihepatectomies, two left trisectionectomies, and one central hepatectomy (resection of segments 4, 5, and 8). In all but one case, TVE was applied during the vascular resection and reconstruction phase, with a median time of 23 min (range 15–73 min). TVE was part of a planned strategy when the parenchymal transection was almost completed and the hepatic venous confluence or the IVC was approached. TVE was well tolerated by all patients and no hemodynamic instability was observed. In patient 16, no TVE was applied but the IVC was clamped laterally and the specimen was resected en bloc with a patch of the anterior wall of the IVC. Eight patients received intraoperative blood transfusions (range 10–32.1 mL/kg). Table 2 shows details on the surgical procedures.

**Table 2:** Intraoperative data

Patient no.	Type of hepatectomy	Type of vascular reconstruction	Total vascular exclusion (min)	Intraoperative blood transfusion	Intraoperative events
1	Central hepatectomy (segments 4, 5, 8 resected)	Reconstruction of the remaining HV (right), closure with direct suture	62	20 mL/kg (total 320 mL)	Hemorrhage after declamping
2	Right trisectionectomy (plus segment I)	Wedge resection of the retrohepatic IVC and closure with direct suture	59	13.3 mL/kg (total 160 mL)	
3	Right trisectionectomy	Reconstruction of the only remaining HV (left), closure with direct suture	73	21.4 mL/kg (total 855 mL)	TVE >60 min
4	Right trisectionectomy (plus segment I)	Reconstruction of the only remaining HV (left) with direct suture	23	–	
5	Right trisectionectomy	Wedge resection of the IVC around the orifice of the right HV, closure with direct suture	40	–	
6	Right hemihepatectomy (middle HV resected)	Wedge resection of the retrohepatic IVC and closure with direct suture	23	–	
7	Right hemihepatectomy (middle HV resected)	Reconstruction of the only remaining HV (left), closure with direct suture	50	–	
8	Right hemihepatectomy	Wedge resection of the IVC around the orifice of the right HV, closure with direct suture	20	10 mL/kg (total 130 mL)	
9	Right trisectionectomy (plus segment I)	Segmental resection of the retrohepatic IVC, end-to-end anastomosis	30	–	
10	Right hemihepatectomy (middle HV resected)	Reconstruction of the only remaining HV (left), closure with direct suture	18	–	
11	Left trisectionectomy	Reconstruction of the only remaining HV (right), closure with direct suture	40	–	
12	Right trisectionectomy (plus segment I)	Reconstruction of the only remaining HV (left), closure with direct suture	20	11.2 mL/kg (total 480 mL)	
13	Right trisectionectomy (plus segment I)	Wedge resection of the retrohepatic IVC and closure with direct suture	23	–	
14	Right trisectionectomy	Reconstruction of the only remaining HV (left), closure with direct suture	20	–	Intraoperative bile leakage (sutured)
15	Right trisectionectomy (plus segment I)	Reconstruction of the only remaining HV (left), closure with direct suture	19	24.9 mL/kg (total 150 mL)	
16	Left trisectionectomy (plus segment I)	Wedge resection of the retrohepatic IVC and closure with direct suture	0	32.1 mL/kg (total 350 mL)	No TVE, lateral clamping of IVC
17	Right trisectionectomy (plus segment I)	Wedge resection of the retrohepatic IVC and closure with direct suture	15	17.9 mL/kg (total 200 mL)	

*HV* hepatic vein, *IVC* inferior vena cava, *TVE* total vascular exclusion

### Strategy and Technique of Vascular Resection and Reconstruction

**Fig. 2:**
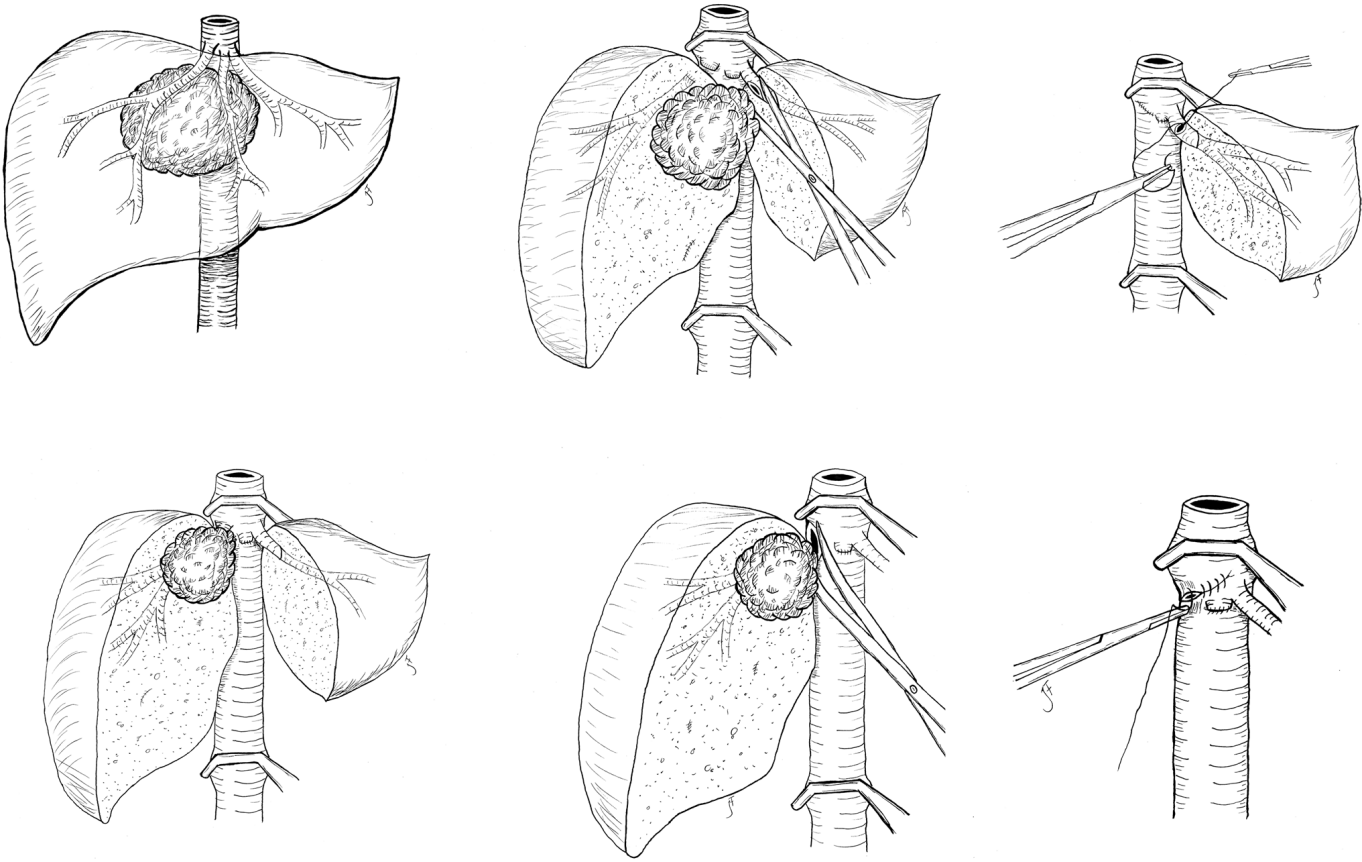
Upper three sketches show type 1 reconstructions of the only remaining hepatic vein. Lower three sketches show type 2 reconstructions of the hepatic vein orifice in the IVC. *IVC* inferior vena cava

**Fig. 3:**
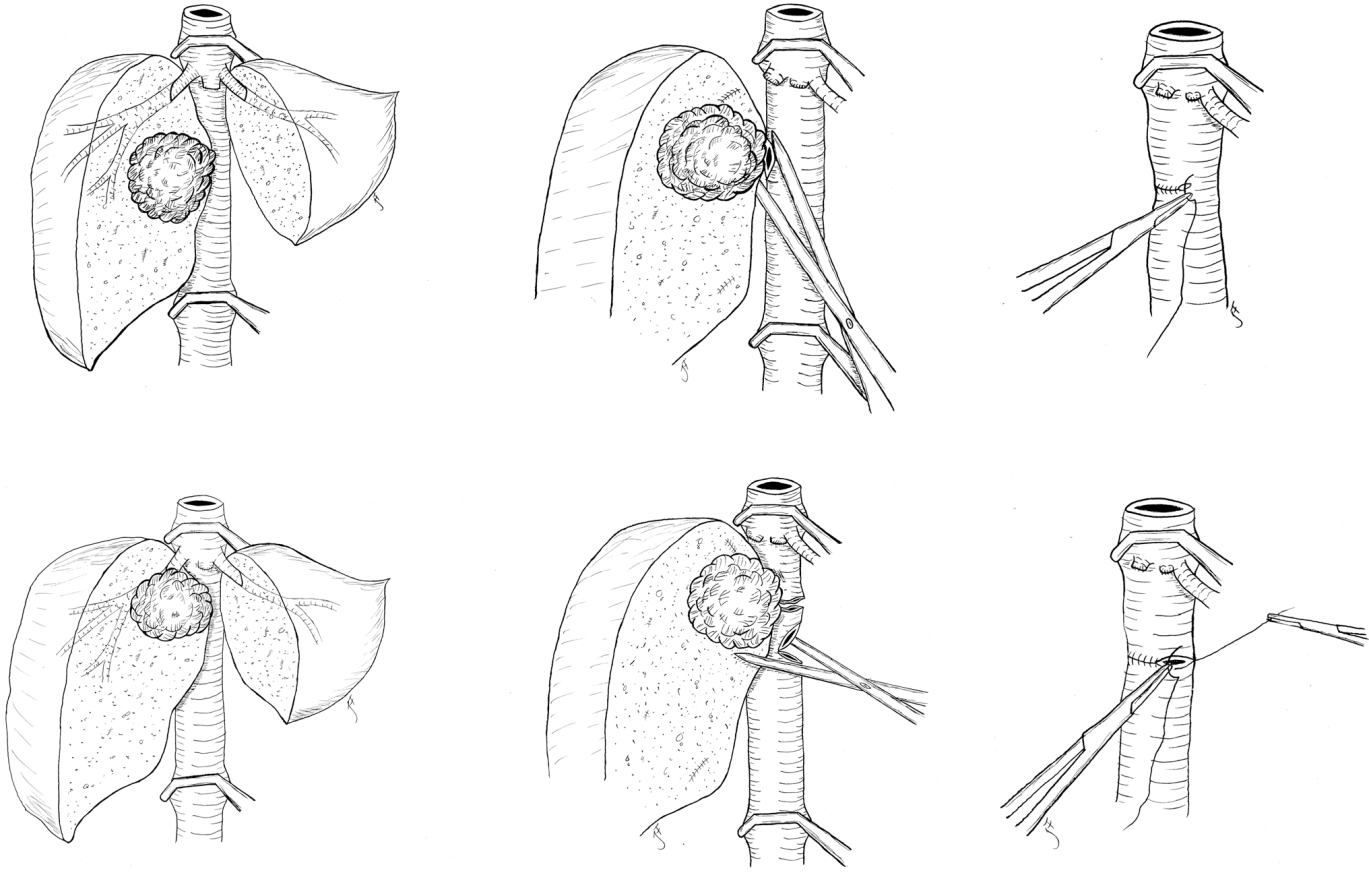
Upper three sketches show type 3 reconstructions of the IVC. Lower three sketches show type 4 reconstructions with segmental resection and end-to-end anastomosis of the IVC. *IVC* inferior vena cava

As described in the Methods section, patients were carefully selected and patency of the remaining HV was an indispensable criteria for the decision to perform liver resection. In all 17 patients, tumor contact to the HV or IVC had been identified preoperatively and surgery was planned with the option of vascular resection and reconstruction. Although we were prepared to perform vascular reconstruction using a prosthesis or patches, the aim was to avoid vascular replacement, and direct vascular reconstruction was possible in all 17 patients. In cases of tumor thrombus in an HV or the IVC, the vessel was opened and the thrombus was removed en bloc with a patch of the vessel wall where the thrombus was found to be adherent. Preoperatively, extension of the thrombus was evaluated by hepatic and cardiac ultrasound and supradiaphragmatic extension was ruled out. The situations encountered in the 17 patients were classified into four different types, as illustrated in Figs. 2 and 3 and described in the following sections.

#### Type 1: Tumor in Close Contact with All Three Hepatic Veins (HVs)

In the first type, the tumor was in contact with all three HVs (often obliterating two of them). These tumors are often either originating from the upper part of segment 1, touching or invading the hepatic venous confluence and the HVs from their posterior side, or centrally located tumors originating from segments 4a, 5, and 8, invading the confluence from the ventral part. The tumor was then carefully dissected off the non-adherent parts of the remaining HV, the vein was opened to rule out more extensive macroscopic tumor invasion, and a small adherent patch of the remaining HV wall was resected. In all nine cases of this type, the HV could be directly reconstructed without a patch, performing a transversal running suture using 6-0 polypropylene (see the upper three sketches in Fig. 2).

#### Type 2: Tumor Invading the Inferior Vena Cava (IVC) Around the Orifice of an HV

In the second type, the tumor was extensively invading the orifice of an HV and the surrounding IVC. In these cases (*n* = 2), a patch of the IVC around the HV orifice was resected en bloc with the specimen and the IVC was directly reconstructed with a transversal running suture using 5-0 Prolene (see the lower three sketches in Fig. 2).

#### Type 3: Tumor Invading the Anterior Wall of the IVC

In the third type, the tumor was invading the anterior or antero-lateral wall of the retrohepatic IVC over a short segment. When a tumor thrombus was present or no dissection plane was found between the liver and the IVC, a patch of the anterior wall of the IVC was resected en bloc with the specimen (in cases of tumor thrombus, the thrombus was removed together with a patch of the IVC wall where the thrombus was adherent). In the five patients undergoing this type of resection, a direct reconstruction of the IVC with a running transversal suture using 5-0 Polypropylene was performed (see the upper three sketches in Fig. 3).

#### Type 4: Tumor Invading the IVC More Than 180°

In patient 9, a more extensive invasion of the retrohepatic IVC required a short segmental resection. Reconstruction with a direct end-to-end anastomosis using two running sutures of 5-0 Polypropylene was possible without tension (see the lower three sketches in Fig. 3).

### Postoperative Outcomes and Follow-Up

There was no 90-day postoperative mortality, and no major complications (CMC ≥3) occurred. Postoperative ultrasounds confirmed hepatic venous drainage and patency of the IVC in all patients. No cases of post-hepatectomy liver failure (PHLF) occurred. Eight patients (47.1%) had minor complications, with the most common being post-hepatectomy hemorrhage (CMC grade II) requiring minor blood transfusions (*n* = 3). Median stay at the intermediate care unit (IMC) was 7 days (range 3–13) and the median length of hospital stay was 10 days (range 8–16).

Macroscopic complete tumor resection and tumor-free vascular margins were achieved in all patients. In four patients, there was no parenchymal or infra-millimetric margin (R1). No local relapse occurred. Patient 10 had a pulmonary relapse and eventually succumbed to the disease 21 months after surgery. The remaining 16 patients were alive without evidence of disease and in first remission after a median follow-up of 44 months (range 19–111). Liver function tests and elastography were normal in all patients at last follow-up, and no cases of late vascular complications were observed. Table 3 shows the postoperative outcomes and follow-up data.

**Table 3:** Postoperative outcomes and follow-up

Patient no.	Postoperative complications (CMC grade)	Days in ICU/IMC	Length of hospital stay, days	Resection margins (parenchymal/vascular)	Relapse	Follow-up
1	None	7	9	R0/R0	No relapse	Alive with NED at 111 months
2	Ileus (II), infection of the central line (II)	8	11	R0/R0	No relapse	Alive with NED at 59 months
3	Post-hepatectomy hemorrhage (II)	5	11	R0/R0	No relapse	Alive with NED at 88 months
4	None	9	12	R1/R0	No relapse	Alive with NED at 111 months
5	None	5	7	R0/R0	No relapse	Alive with NED at 46 months
6	None	8	10	R0/R0	No relapse	Alive with NED at 101 months
7	Urinary tract infection (II)	7	9	R0/R0	No relapse	Alive with NED at 35 months
8	Urinary tract infection (II), pneumonia (II)	7	11	R0/R0	No relapse	Alive with NED at 53 months
9	Post-hepatectomy hemorrhage (II)	13	16	R1/R0	No relapse	Alive with NED at 26 months
10	None	6	9	R0/R0	Pulmonary relapse	DOD at 21 months
11	None	7	10	R1/R0	No relapse	Alive with NED at 41 months
12	Bacteremia without sepsis (II)	7	9	R0/R0	No relapse	Alive with NED at 51 months
13	Pneumonia (II)	6	12	R0/R0	No relapse	Alive with NED at 41 months
14	Post-hepatectomy hemorrhage (II)	3	9	R1/R0	No relapse	Alive with NED at 29 months
15	None	8	13	R0/R0	No relapse	Alive with NED at 26 months
16	None	5	12	R0/R0	No relapse	Alive with NED at 30 months
17	None	5	8	R0/R0	No relapse	Alive with NED at 19 months

Roman numerals in parentheses indicate the grade of complication according to the CMC

*CMC* Clavien–Madadi classification, *ICU* intensive care unit, *IMC* intermediate care unit, *NED* no evidence of disease, *DOD* death of disease

## Discussion

To our knowledge, this study presents the largest single-center series of pediatric patients who underwent major hepatectomy with HV or IVC reconstruction for malignant liver tumors. The results of this study provide insights into the safety and feasibility of such complex procedures in the pediatric population. Our findings demonstrate that with meticulous preoperative planning and surgical experience, extended liver resections involving vascular reconstruction can be performed with excellent short- and long-term outcomes, thus avoiding the need for liver transplantation in carefully selected patients.

### Patient Selection and Criteria for Resection versus Transplantation

Rigorous patient selection based on a profound knowledge of tumor characteristics plays a pivotal role in determining the feasibility of liver resection with vascular reconstruction in pediatric liver surgery.^15^ By adhering to our selection criteria, we ensured that resection is only pursued in cases where organ preservation is viable without excessive surgical risks. The absence of major complications and a minor morbidity rate of 47% in our series of complex liver resection compares favorably with the morbidity after pediatric liver transplantation, which is reported to be higher than 50% in most studies.^16,17^ At the same time, we consider transplantation an invaluable therapy for truly unresectable pediatric liver tumors, and our center provides the infrastructure and the expertise to perform liver transplantation whenever indicated. We emphasize that the decision for resection was based solely on oncologic and patient safety reasons and was not driven by either limited access to suited organs or surgeons’ ambition to perform complex hepatectomy. On the contrary, as previously mentioned, our patients are granted priority access to deceased donor organs, and liver transplantation is performed by the same surgeons as the liver resections at our center, suggesting that there should be no underlying bias for resection in our series. It is our conviction and experience that if resection is feasible, it should be the first choice for children with locally advanced liver tumors, as oncologic outcomes are equal, and the long-term negative consequences of liver transplantation can be avoided in these patients. In 2 of the 17 patients in our series, liver transplantation was initially the intended approach, but our results suggest that an organ-preserving approach should be strongly considered in cases where partial resection and vascular reconstruction are feasible. Moreover, transplantation may be associated with more long-term drawbacks, including lifelong immunosuppression and the risk of late graft complications such as biliary stenosis or rejection, which can eventually lead to graft loss and the need for retransplantation. Looking at the existing evidence, only a few studies with low caseloads explored the option of liver resection in pediatric liver tumors with hepatic venous confluence and IVC involvement. Uchida et al. described their strategy in nine children with hepatoblastoma and major vascular involvement; liver transplantation was performed in five of these patients, and extended liver resection was performed in four patients.^18^ Postoperative morbidity occurred in two patients who underwent resection, and one patient experienced liver recurrence. All patients were alive at last follow-up. Fonseca et al. demonstrated their strategy to accept minimal margins and IVC reconstruction in five children with locally advanced hepatoblastoma.^19^ No local relapse was observed and all five patients were alive at last follow-up. In the study by Fuchs et al. of 27 children with locally advanced hepatoblastoma (POST-TEXT III or IV), six patients underwent vascular reconstruction, with no local relapse reported in the study cohort.^1^ Case reports of liver resection with vascular reconstruction in children also reported favorable outcomes,^9,10^ however larger series are lacking. Our results show that tumor contact to all three HVs, or involvement of the IVC, should not routinely be considered a contraindication to liver resection in pediatric liver tumors. The applied patient selection criteria and our surgical strategy have allowed us to successfully perform extended resections in patients who might have been, or in fact had been, considered for liver transplantation, emphasizing the importance of tailored, case-by-case decision making in locally advanced pediatric liver tumors.

### Impact of Resection Margins

Accepting minimal resection margins (<1 mm), as we do in selected cases, seems to be a reasonable strategy for most pediatric liver tumors, and also from an oncological standpoint. Previous studies on the role of resection margins in hepatoblastoma, especially SIOPEL data of 431 children undergoing surgery for hepatoblastoma, suggested that R1 resections neither increase the risk of recurrence nor negatively influence the overall outcome.^20,21^ At the same time, we strongly emphasize that the resection line should always respect tumor tissue and never pass through the tumor, and the tumor capsule should not be injured. In cases of actual vascular tumor invasion, we aimed for a macroscopical complete resection en bloc with the infiltrated portion of the vessel wall. In our series, no local recurrence was observed by applying this strategy. To fully elucidate the impact of resection margins, future studies should further investigate the role of parenchymal and vascular margins in pediatric liver tumors, including biological aspects of tumor subtypes, to establish evidence-based guidelines for the surgical strategy.

### Technical Feasibility, Safety, and Oncologic Outcomes of Vascular Reconstruction in Children Undergoing Hepatectomy

We performed vascular reconstructions in 17 children, with no 90-day mortality, absence of major complications, and no local relapse, with an overall survival rate of 94% at a median follow-up of 44 months. Hepatic outflow was preserved in all patients, as objectified by postoperative Doppler ultrasound. We therefore showed that this approach is not only technically feasible but, more importantly, safe and oncologically effective. The planned application of a TVE, without major hemodynamic instability, and the absence of PHLF are further important aspects of the demonstrated technique. While TVE is associated with increased morbidity in adult patients,^22–24^ our results show that it can be safely applied in children. The higher blood volume per weight, as well as the higher percentage of supradiaphragmatic circulating blood volume in children compared with adults, may explain the better hemodynamic tolerance of TVE in children.^25^ Moreover, children are much less frequently affected by cardiovascular disease compared with adults, which may further explain the better tolerance of TVE. Moreover, our experience indicates that pediatric patients tolerate extensive liver resections exceptionally well without relevant risks of PHLF, as shown in one of our groups’ previous publications.^26^

### Limitations and Future Directions

The retrospective nature of the data and the small sample size of patients undergoing vascular reconstruction limits the generalizability of our findings. Additionally, while no recurrences or mortalities were observed in the medium term, longer follow-up is required to ensure that these oncologic and liver function outcomes remain durable over time. Future multicenter studies focusing on surgical strategies could help validate these findings and further refine selection criteria for extended hepatectomy with vascular resection in pediatric patients.

## Conclusion

This study demonstrates that extended liver resections with concurrent vascular reconstruction for tumors involving the hepatic venous confluence and the IVC can be safely performed in children. This organ-preserving approach offers an alternative to liver transplantation in selected patients, with excellent short- and long-term outcomes. These results underscore the importance of experienced surgical teams in the treatment of children with liver tumors and the need for treatment strategies tailored to pediatric patients to optimize outcomes for children with complex liver tumors.

## Supplementary Information

Below is the link to the electronic supplementary material.

Supplementary file1 (DOCX 33 KB)

## Data Availability

The datasets used and/or analyzed for the current study are available from the corresponding author upon reasonable request.
